# Endothelial Progenitor Cell Therapy for Fracture Healing: A Dose-Response Study in a Rat Femoral Defect Model

**DOI:** 10.1155/2023/8105599

**Published:** 2023-03-09

**Authors:** David J. Ramnaraign, Charles Godbout, Kalter Hali, Christian Hegner, Brent D. Bates, Sarah Desjardins, Jonathan Peck, Emil H. Schemitsch, Aaron Nauth

**Affiliations:** ^1^Keenan Research Centre for Biomedical Science, Unity Health Toronto (St. Michael's Hospital), University of Toronto, Toronto, Ontario, Canada; ^2^Division of Orthopaedic Surgery, Department of Surgery, Unity Health Toronto (St. Michael's Hospital), University of Toronto, Toronto, Ontario, Canada; ^3^Department of Surgery, Western University, London, Ontario, Canada

## Abstract

Endothelial progenitor cell (EPC) therapy has been successfully used in orthopaedic preclinical models to heal bone defects. However, no previous studies have investigated the dose-response relationship between EPC therapy and bone healing. This study aimed to assess the effect of different EPC doses on bone healing in a rat model to define an optimal dose. Five-millimeter segmental defects were created in the right femora of Fischer 344 rats, followed by stabilization with a miniplate and screws. Rats were assigned to one of six groups (control, 0.1 M, 0.5 M, 1.0 M, 2.0 M, and 4.0 M; *n* = 6), receiving 0, 1 × 10^5^, 5 × 10^5^, 1 × 10^6^, 2 × 10^6^, and 4 × 10^6^ EPCs, respectively, delivered into the defect on a gelatin scaffold. Radiographs were taken every two weeks until the animals were euthanized 10 weeks after surgery. The operated femora were then evaluated using micro-computed tomography and biomechanical testing. Overall, the groups that received higher doses of EPCs (0.5 M, 1.0 M, 2.0 M, and 4.0 M) reached better outcomes. At 10 weeks, full radiographic union was observed in 67% of animals in the 0.5 M group, 83% of animals in the 1.0 M group, and 100% of the animals in the 2.0 M and 4.0 M groups, but none in the control and 0.1 M groups. The 2.0 M group also displayed the strongest biomechanical properties, which significantly improved relative to the control and 0.1 M groups. In summary, this study defined a dose-response relationship between EPC therapy and bone healing, with 2 × 10^6^ EPCs being the optimal dose in this model. Our findings emphasize the importance of dosing considerations in the application of cell therapies aimed at tissue regeneration and will help guide future investigations and clinical translation of EPC therapy.

## 1. Introduction

Despite the substantial regenerative potential of bone, compromised fracture healing remains a major challenge for orthopaedic surgeons with nonunion prevalence reaching over 10% in cases of femoral or tibial fractures [1, 2]. Affected patients often require further invasive interventions, with the concurrent risk of adverse events and complications, adding to the length of recovery. As a result, fracture nonunion can impact the functional outcomes and quality of life of patients and increase the financial burden on the healthcare system [3–5]. The current gold standard treatment relies on autologous iliac crest bone graft (AICBG), which represents a histocompatible scaffold containing both osteogenic cells and osteoinductive growth factors [6]. However, AICBG is not recommended as a stand-alone treatment for large segmental defects. Moreover, the amount of graft material available is limited, and the harvesting procedure can lead to complications at the donor site and produce substantial morbidity [7], encouraging investigations into new avenues.

In this context, endothelial progenitor cells (EPCs) have become promising therapeutic agents due to their angiogenic/vasculogenic properties [8]. Indeed, blood supply is crucial to initiate and support bone regeneration and, accordingly, poor vascularization has been associated with nonunion [9]. Within recent years, we have demonstrated consistent success using EPCs to repair critical-size defects in the femora of rats [10–13]. Since there is no consensus regarding the optimal dose of EPCs for tissue regeneration in the literature, the number of implanted cells used in our previous studies (1 million cells) was arbitrary to some extent. To progress towards clinical translation, it is essential to thoroughly characterize and optimize EPC-based treatment and address this limitation.

Consequently, this study evaluated the impact of different doses of EPCs on critical-size bone defect healing in a rat model. Our main objectives were to determine an optimal number of cells that would yield the greatest therapeutic benefit and determine if a dose-response relationship exists in the use of EPCs for bone healing. Therefore, we created six different treatment groups receiving increasing doses of EPCs, ranging from 0 to 4 million cells, implanted into a surgically created bone defect in a rat femur. We evaluated the bone healing response with radiographs, micro-computed tomography (micro-CT), and biomechanical testing in order to identify a dose-response relationship between EPCs and bone healing.

## 2. Materials and Methods

### 2.1. Animals

Male Fischer-344 rats (250–300 g; Charles River Laboratories, Kingston, NY) were used in this study as an in vivo model of segmental bone defect as well as a source for bone marrow derived progenitor cells. Throughout the protocol, food and water were provided ad libitum to rats and a 12 h-light/12 h-dark cycle was maintained in the housing room. All animal procedures were approved by the Institutional Animal Care Committee at St. Michael's Hospital and conducted according to its guidelines.

### 2.2. Isolation and Culture of Endothelial Progenitor Cells

Donor rats were anesthetized by inhalation of isoflurane (5% in oxygen (2 L/min)) and then euthanized via cervical dislocation. We excised the femora and tibiae and collected bone marrow by flushing the medullary canals with sterile PBS (without Ca^2+^/Mg^2+^; Gibco, Thermo Fisher Scientific, Waltham, MA). The resulting mixture was centrifuged for 10 min at 360*g*. We subsequently aspirated the supernatant and resuspended the pellet in Endothelial Basal Medium-2 (EBM-2; Lonza, Walkersville, MD) supplemented with the EGM-2 MV SingleQuots kit (Lonza). The cell suspension obtained from each animal was then divided evenly and transferred into two T75 culture flasks (Sarstedt, Montreal, Canada) previously coated with fibronectin (10 *μ*g/mL in PBS; EMD Millipore, Etobicoke, Canada). Cells were then incubated under standard conditions (37°C, 5% CO_2_) for 7-8 days until surgical implantation, with a change of culture medium every two days. Using this technique, we have previously demonstrated that the isolated cell population displays a spindle-shaped morphology characteristic of EPCs after 7-8 days in culture, forms tube-like structures when seeded on Matrigel, and stains double positive for uptake of acetylated low-density lipoprotein and binding of *Ulex europaeus* agglutinin-I [11].

### 2.3. Experimental Design and Surgical Procedures

We assigned 36 rats to one of six groups (control, 0.1 M, 0.5 M, 1.0 M, 2.0 M, and 4.0 M), receiving 0, 1 × 10^5^, 5 × 10^5^, 1 × 10^6^, 2 × 10^6^, and 4 × 10^6^ EPCs, respectively (*n* = 6). The rats were maintained under general anesthesia (2.5% isoflurane in oxygen (2 L/min)) during the procedures. The right leg was shaved, followed by application of isopropyl alcohol and povidone-iodine. As previously described, we exposed the right femur through a lateral approach under aseptic conditions [10]. Then, we created a 5 mm mid-diaphyseal defect with an oscillating saw by performing two osteotomies under constant saline irrigation. We stabilized the bone with a straight plate (2.5 cm × 3.8 mm × 0.8 mm; Synthes, Mississauga, Canada) and four 1.5 mm diameter cortical screws (two 8.0 mm and two 6.0 mm long; Synthes). After being harvested and resuspended in medium, EPCs had been seeded at least 30 min beforehand onto a sterile gelatin scaffold (approximately 5 × 5 × 5 mm; Gelfoam; Pfizer, Kirkland, Canada), at a dose corresponding to the treatment group assigned to each animal. Scaffolds prepared for the control group were only impregnated with culture medium. After placing the designated scaffold into the bony defect, we closed the muscle layers and skin with absorbable sutures (Vicryl 5.0; Johnson and Johnson, Markham, Canada). Postoperatively, animals were housed in individual cages where full weight-bearing and unrestricted movement were immediately allowed. Pain management was achieved by preoperative and postoperative (twice daily for 48 h) subcutaneous administration of buprenorphine hydrochloride (0.05 mg/kg; Temgesic; Indivior, Berkshire, UK). No anti-inflammatory medication was used.

### 2.4. Radiographic Assessment

To examine the progression of bone healing, serial anteroposterior radiographs of the operated femur of each animal were taken every two weeks until euthanasia (OEC Medical Systems Series 9800; GE Healthcare, Mississauga, Canada). A constant tube-to-cassette distance was maintained, with a constant voltage of 64 kVp and a current of 2 mAs. In a blinded and independent fashion, two orthopaedic surgeons (JP, AN) evaluated each radiograph according to bone filling of the defect and callus density (Table 1) [11]; the individual average score was recorded. Reviewers also assessed the union status, defined as either full union, partial union, or nonunion. In cases of disagreement (e.g., full union vs. partial union), the most conservative assessment was used.

### 2.5. Euthanasia and Micro-Computed Tomography Analysis

Ten weeks after surgery, we euthanized all animals via cardiac injection of T-61 (Merck, Kirkland, Canada) under general anesthesia. The operated femora were excised and transferred into a 10% neutral buffered formalin solution after removing the plate and screws.

Then, we performed micro-CT analysis on all operated femora to quantify bone formation at the defect site (*µ*CT 40; Scanco Medical, Brüttisellen, Switzerland). Following a protocol previously described [11], the same scanning parameters (70 kVp, 114 *µ*A, and 8 *µ*M voxel) and threshold value for bone detection (263) were applied to all samples. The resulting 2D transverse images were reconstructed to provide vertical views of the femur and defect. The region of interest (ROI) was defined as a rectangle covering the defect site, using the recognizable sites of the original osteotomies and the widest edges of the native bone as boundaries. The same ROI was applied to all slices within the confines of the native cortices. The following outcomes related to our ROI are reported: bone volume (BV; mm^3^), bone volume ratio (BV/total volume (TV)), trabecular number (Tb.N; 1/mm), and trabecular separation (Tb.Sp, mm).

### 2.6. Biomechanical Testing

We tested the operated femora under a torsional load using an MTS Bionix 858 (MTS Systems, Eden Prairie, MN). In brief, each bone was potted in polymethylmethacrylate contained within hexagonal locknuts and screwed carefully into place along the longitudinal axis of the testing device. A gauge length of 20 mm was maintained. The femora were loaded at a constant rate of 1° per second and the torque was measured until failure or until a displacement of 40° was reached. Across the generated torque-angular displacement curve, we calculated successive slopes over a displacement of 1°. The maximum stiffness represented the highest slope within the linear portion of the curve. We then identified the first following slope at least 10% lower than the maximum stiffness [14]. The yield point was defined as the torque measured at the end of this section of the curve. The highest torque value recorded was considered as the maximum torque.

Operated specimens that did not show any bridging of the defect, based on radiographic assessment and micro-CT imaging, were assigned a zero value. Indeed, when two bone segments are connected through fibrous tissue only, sample preparation is highly variable, and the resulting curve is generally erratic and without a clear breaking point. All these elements make the analysis unreliable when there is a defect without any bony bridging. However, samples were tested if any uncertainty remained regarding the bridging of the defect.

### 2.7. Statistical Analysis

To compare animal weights, radiographic scores at each time point, and micro-CT values, we performed a one-way analysis of variance (ANOVA) followed by a post hoc analysis using Tukey's HSD test. Biomechanical results were compared among groups using a Kruskal–Wallis test followed by a post hoc analysis using Dunn's test. A *p* value equal to or less than 0.05 was considered significant. These statistical analyses were performed using RStudio (Version 2022.02.3). For radiographic assessment, agreement between raters was evaluated based on a weighted kappa coefficient for the union status, and an intraclass correlation coefficient (ICC; two-way mixed, absolute agreement, and single measure) for radiographic scoring (SPSS, v23; IBM, Armonk, NY) [15, 16].

## 3. Results

### 3.1. Animals

All animals (36/36) reached the endpoint of the study and contributed to radiographic, micro-CT, and biomechanical results. There were no statistically significant differences in animal weights between the groups on the day of the surgery nor on the day of euthanasia on week 10 (*p* > 0.05; Supplementary Table 1).

### 3.2. Radiographic Assessment

For the radiographic assessment, agreement between raters regarding the union status was considered almost perfect (weighted kappa coefficient = 0.848). Similarly, the inter-rater reliability was excellent for radiographic scoring (ICC = 0.931).

**Figure 1:**
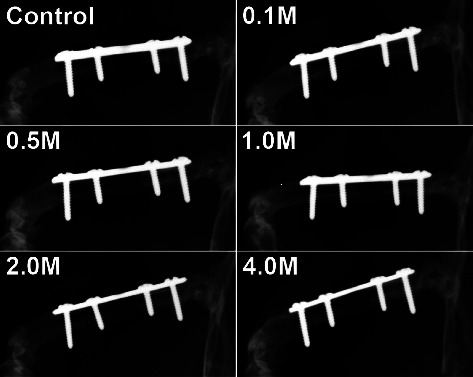
Anteroposterior radiographic images at 10 weeks following endothelial progenitor cell (EPC) implantation into the defect site. Control refers to the animals that received no EPCs; 0.1 M, 0.5 M, 1.0 M, 2.0 M, and 4.0 M refer to the animals that received 1 × 10^5^, 5 × 10^5^, 1 × 10^6^, 2 × 10^6^, and 4 × 10^6^ EPCs, respectively.

Over the postoperative course, full radiographic union was reached in 67% (4/6) and 83% (5/6) of animals in the 0.5 M and 1.0 M groups, respectively, and in 100% of the animals in the 2.0 M and 4.0 M groups (Figure 1, Table 2). In contrast, none of the operated femora demonstrated full union in the control and 0.1 M groups.

**Figure 2:**
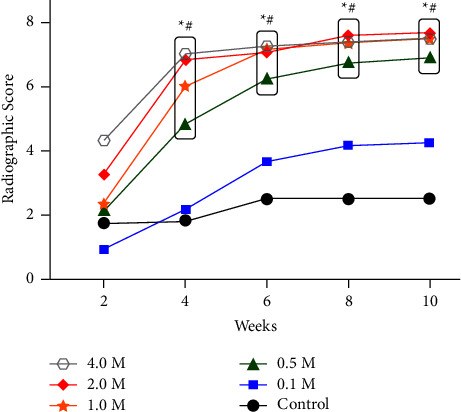
Mean radiographic scores of new bone formation across the different animal groups. Control refers to the animals that received no endothelial progenitor cells (EPCs); 0.1 M, 0.5 M, 1.0 M, 2.0 M, and 4.0 M refer to the animals that received 1 × 10^5^, 5 × 10^5^, 1 × 10^6^, 2 × 10^6^, and 4 × 10^6^ EPCs, respectively. ^*∗*^denotes a significant difference between groups surrounded by the box (0.5 M, 1.0 M, 2.0 M, and 4.0 M) and the control group (*p* ≤ 0.05). ^#^denotes a significant difference between groups surrounded by the box (0.5 M, 1.0 M, 2.0 M, and 4.0 M) and the 0.1 M group (*p* ≤ 0.05).

At week 2, animals in the 4.0 M group had the highest mean radiographic score. However, no statistical differences were noted between any of the groups (*p* > 0.05; Figure 2, Supplementary Table 2). From weeks 4 to 10, mean radiographic scores were significantly higher in the 0.5 M, 1.0 M, 2.0 M, and 4.0 M groups than in the control and 0.1 M groups (*p* ≤ 0.05). There were no statistically significant differences in radiographic scores between the 0.5 M, 1.0 M, 2.0 M, and 4.0 M groups at any time points throughout the study (*p* > 0.05).

### 3.3. Biomechanical Testing

Bone specimens collected 10 weeks after surgery were tested under torsional load to assess their mechanical properties. Two specimens (one in each of the 0.5 M and 1.0 M groups), considered to have reached partial union, produced a curve similar to nonunion cases and were considered as such, receiving zero values.

**Figure 3:**
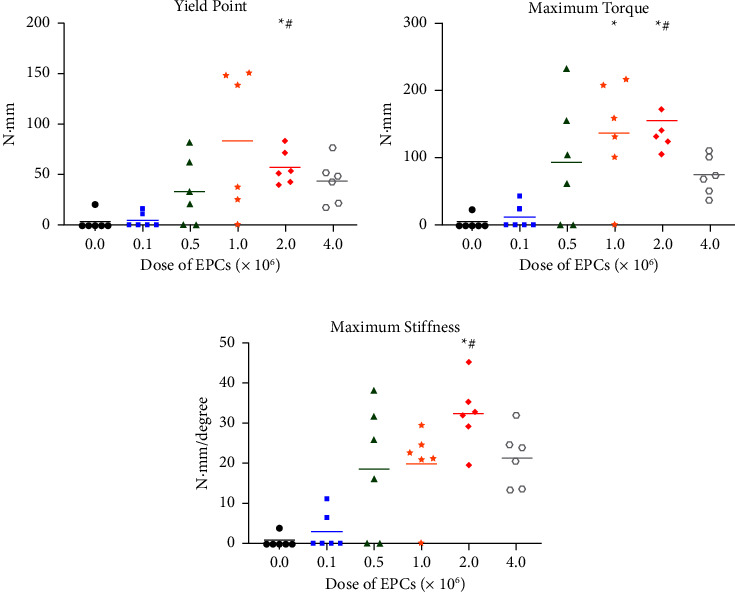
Biomechanical properties of the operated femora across the different animal groups: (a) yield point, (b) maximum torque, and (c) maximum stiffness. Each symbol represents an individual outcome. The horizontal lines represent the mean for each group. Control refers to the animals that received no endothelial progenitor cells (EPCs); 0.1 M, 0.5 M, 1.0 M, 2.0 M, and 4.0 M refer to the animals that received 1 × 10^5^, 5 × 10^5^, 1 × 10^6^, 2 × 10^6^, and 4 × 10^6^ EPCs, respectively. ^*∗*^denotes a significant difference from the control group (*p* ≤ 0.05). ^#^denotes a significant difference from the 0.1 M group (*p* ≤ 0.05).

In general, mechanical outcomes distinctively improved with doses of 5 × 10^5^ EPCs or higher (Figure 3), which is consistent with the radiographic assessment. The yield point, maximum torque, and maximum stiffness recorded in the 2.0 M group were significantly higher than in the control and 0.1 M groups (*p* ≤ 0.05; Figures 3(a)–3(c)). Specimens from the 1.0 M group also showed a statistically higher maximum torque than the control group (*p* ≤ 0.05; Figure 3(b)). Interestingly, when testing specimens from the 4.0 M group, mechanical results appeared to decline, particularly in relation to the maximum torque and stiffness (Figures 3(b) and 3(c)). Indeed, the mean torque and stiffness values reached 157.2 N·mm and 32.4 N·mm/degree, respectively, in the 2.0 M group but decreased to 74.5 N·mm and 21.2 N·mm/degree in the 4.0 M group. However, these changes did not reach statistical significance (*p* > 0.05).

### 3.4. Micro-Computed Tomography Analysis

**Figure 4:**
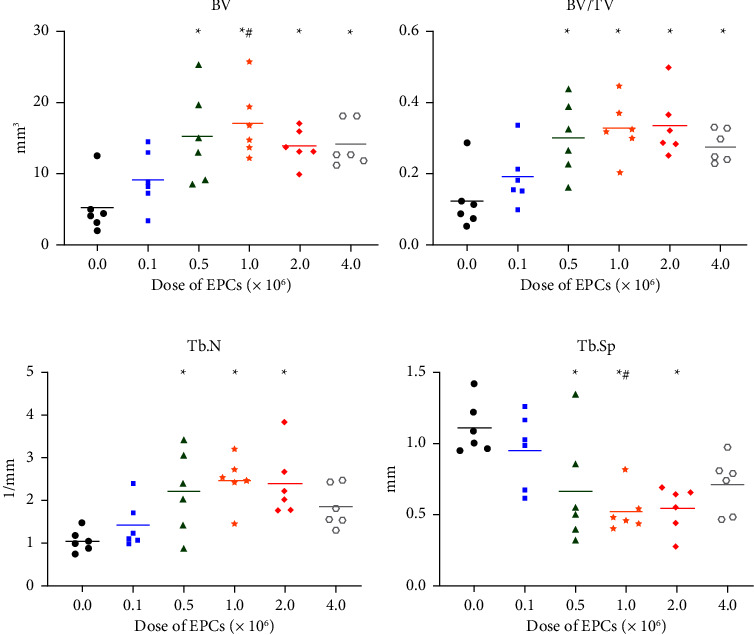
Micro-computed tomography evaluation of new bone formation across the different animal groups: (a) BV refers to bone volume (mm^3^), (b) BV/TV refers to bone volume/total volume, (c) Tb.N refers to trabecular number (1/mm) and (d) Tb.Sp refers to trabecular separation (mm). Each symbol represents an individual outcome. The horizontal lines represent the mean for each group. Control refers to the animals that received no endothelial progenitor cells (EPCs); 0.1 M, 0.5 M, 1.0 M, 2.0 M, and 4.0 M refer to the animals that received 1 × 10^5^, 5 × 10^5^, 1 × 10^6^, 2 × 10^6^, and 4 × 10^6^ EPCs, respectively. ^*∗*^denotes a significant difference from the control group (*p* ≤ 0.05). ^#^denotes a significant difference from the 0.1 M group (*p* ≤ 0.05).

Micro-CT scanning, reconstruction, and analysis of the operated samples provided in-depth information on the topographical features of the defect site (Figure 4). Similar to radiographic and biomechanical outcomes, parameters improved with doses of at least 5 × 10^5^ EPCs. When compared with the control group, specimens from the 0.5 M, 1.0 M, and 2.0 M groups demonstrated significantly higher BV, BV/TV, and Tb.N, along with lower/improved Tb.Sp (*p* ≤ 0.05; Figures 4(a)–4(d)). In addition, the BV and Tb.Sp of animals in the 1.0 M group were significantly improved compared with animals in the 0.1 M group (*p* ≤ 0.05). In contrast, the 4.0 M group differed from the control values only in regard to BV and BV/TV (*p* ≤ 0.05).

**Figure 5:**
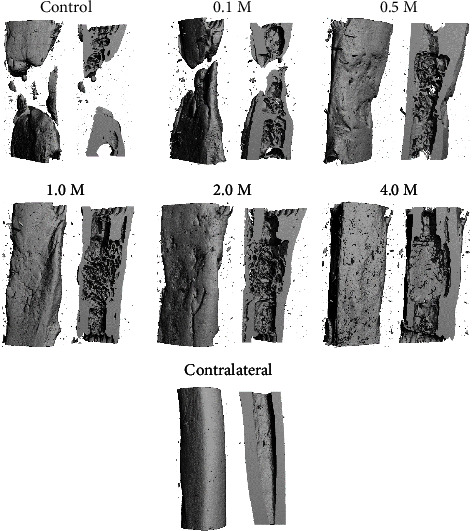
3D reconstruction of a representative femur from each group created based on micro-computed tomography data. For each representative femur, the image on the left provide an outside view of the bone, and the image on the right show the bone midsection. Control refers to the animals that received no endothelial progenitor cells (EPCs); 0.1 M, 0.5 M, 1.0 M, 2.0 M, and 4.0 M refer to the animals that received 1 × 10^5^, 5 × 10^5^, 1 × 10^6^, 2 × 10^6^, and 4 × 10^6^ EPCs, respectively. Contralateral refers to the non-operated, contralateral femur.

The trend observed with biomechanical testing was supported by micro-CT outcomes, in particular for BV/TV, Tb.N, and Tb.Sp (Figures 4(b)–4(d)). Mean values showed a gradual progression, peaking in the 1.0 M or 2.0 M groups, followed by decreased BV/TV and Tb.N and an increased/inferior Tb.Sp in the 4.0 M group. These variations of trabecular parameters appear to be consistent with 3D models created from micro-CT data (Figure 5). Indeed, mid-sectional images of specimens from the 4.0 M group show a relatively empty medullary space, especially when compared with the trabecular bone structure developed in healed specimens of the 1.0 M and 2.0 M groups.

## 4. Discussion

The healing of large bone defects and nonunions is a well-documented clinical issue that is still investigated from a myriad of perspectives. Recently, promising preclinical data have prompted interest in EPC therapy to augment bone healing [10–13]. However, to our knowledge, there has been no report on a dose-response relationship between EPC therapy and bone healing. Therefore, this study aimed to assess the effect of different EPC doses on bone healing. The results of this study demonstrate that higher doses of EPCs result in improved union rates, higher quality bone, and greater biomechanical stability when compared to a control group and a low dose of cells (1 × 10^5^). Furthermore, our findings demonstrate no added benefit of increasing the EPC dose beyond 2 × 10^6^ cells as demonstrated by the lack of bone quality improvement at a higher dose.

Radiographic evaluation of bone healing 10 weeks following the defect surgery demonstrated full union in 67% of animals in the 0.5 M group, 83% of animals in the 1.0 M group, and 100% of the animals in the 2.0 M and 4.0 M groups, with none of the animals in the control or 0.1 M groups reaching union. Nevertheless, the quantitative analysis of bone formation and biomechanical properties are also important to consider when assessing the effectiveness of cell therapy on bone healing. In comparison with the control group, animals treated with higher doses of EPCs had significantly increased BV and BV/TV, which have been shown to be good predictors of torsional strength and rigidity [17]. Under biomechanical testing, the 2.0 M group displayed a significantly higher yield point, maximum torque, and maximum stiffness than the control and 0.1 M groups. Importantly, no further improvement was achieved by increasing the dose to 4 × 10^6^ cells. In fact, the 4.0 M group showed a decrease in average BV/TV and Tb.N, alongside an increase in Tb.Sp when compared with the 1.0 M and 2.0 M groups. These results might indicate a more advanced stage in the healing process with reforming of the medullary canal and a predominantly cortical bone structure in animals in the 4.0 M group. The decrease in the trabecular bone observed in the medullary space on the 3D models of the 4.0 M group might also support this hypothesis. However, if this was the case, the femora isolated from these animals would presumably display improved biomechanical properties compared with other groups as the cortical bone can withstand higher stress prior to failure than trabecular bone [18]. However, biomechanical results obtained from the 4.0 M group demonstrated a decrease in mean values when compared with the 2.0 M group. As such, it is unlikely that the animals in the 4.0 M group were at a more advanced stage in the bone-healing process. Taken together, these results suggest that 2 × 10^6^ cells is the optimal dose in this model, and that a plateau or even a decrease in the effectiveness of EPC therapy occurs at a higher dose.

Consistent with our results, higher doses of bone marrow mononuclear cells (1 × 10^7^ cells) did not lead to higher bone formation and bone mineral density than lower doses (1 × 10^6^ and 5 × 10^6^ cells) in a segmental femoral defect in a rat model [19]. However, these findings appear inconsistent with a meta-analysis of studies involving large animal models treated with stem cells, which concluded that transplantation of a higher number of cells (≥ 10^7^) increases new bone formation to a greater degree [20]. In addition, a higher dose (3 × 10^7^ cells) of adipose-derived adult stem cells led to higher new bone volumes compared with lower doses (3 × 10^5^ and 3 × 10^6^ cells) in a rabbit model [21]. Discrepancies in the dose-response relationships observed might be due to various factors, including the cell types and animal models used. Alternatively, it is possible that studies reporting no plateau in effectiveness at higher cell doses did not include sufficiently high doses to reach a potential plateau or decrease in bone healing.

Preclinical studies assessing the effect of cell therapy on heart disease have yielded similar inconsistent findings, with higher doses demonstrating both improvement [22, 23] and no improvement [24, 25] in heart function in comparison with lower doses. The explanations proposed for the lack of increased efficacy at higher cell doses included the following: (i) the competition for limited nutrient resources in highly stressed environments is more intense at higher doses than at lower doses of cells, leading to limited cell survival and/or function and (ii) a larger number of cells may elicit a more profound inflammatory or immune response that accelerates clearance of cells [24]. The latter is not likely to apply to our study considering that syngeneic rats were used, allowing for transplantation of cells from one animal to another without evoking an immune response. As such, it is possible that the increased competition for nutrients at the defect site may have led to decreased numbers and/or function of EPCs in the 4.0 M compared with the 2.0 M group.

Whereas our results suggest that a dose of 2.0 × 10^6^ cells is optimal, the 0.5 M and 1.0 M groups also reached successful outcomes. In the 1.0 M group in particular, full radiographic union was observed in 5/6 animals with the remaining animal showing partial union. Across five previous studies using similar methods and the same EPC dose, a total of 31 out of 32 rats displayed full union after 10 weeks [10–13, 26]. Moreover, in the present study and others, micro-CT and biomechanical results confirmed the effectiveness of a dose of 1 × 10^6^ cells [10, 11, 13]. From a clinical perspective, this could mean that the benefits of EPC therapy for bone healing are not narrowly limited around an optimal dose; this approach would remain valuable even when applied at suboptimal doses.

In this study, EPCs were implanted acutely, immediately after surgically creating the defect, and therefore in the inflammatory stage of the healing cascade. Clinically, we hypothesize that EPC therapy will evolve as a tool used in a delayed fashion to augment healing in fractures that are not expected to achieve union naturally. This would likely take place after the initial inflammatory phase has passed and is why we consider our acute treatment a potential limitation. However, previous work has shown that EPCs have similar healing capabilities whether delivered acutely or three weeks following bone injury, once the initial inflammatory stage has passed [11]. Furthermore, while previous studies have characterized the cell population obtained through our cell isolation and culture technique, we recognize that the cell population obtained is unlikely to represent a pure EPC population. It is likely that small numbers of other cell types (i.e., macrophages or mesenchymal stem cells) were present, potentially impacting healing outcomes. Finally, this study was not designed to elucidate the mechanism behind the dose-response relationship between EPC therapy and bone healing, which limits the explanations of our results. Future studies should aim to investigate the cellular environment of bone healing when treated with different doses of EPCs to gain a deeper understanding of the processes involved.

## 5. Conclusion

Our findings help reinforce and validate the use of EPCs to promote healing in bone defects. Importantly, this study defined a dose-response relationship between EPC therapy and bone healing in the bone defect model used: higher doses (5 × 10^5^ to 4 × 10^6^ cells) generally led to better outcomes than the control group (no cells) or a lower dose (1 × 10^5^ cells), with 2 × 10^6^ cells appearing to be the optimal dose. Having a defined optimal dose is a critical step to guide future research and progress towards clinical translation. Clinically, our results also emphasize the importance of dosing considerations in the application of cell therapies aimed at tissue regeneration.

## Figures and Tables

**Table 1:** Radiographic scoring system.

Defect filling (%)	Callus density	Score
0	N/A	0

1–25	Low	1
	High	2

26–50	Low	3
	High	4

51–75	Low	5
	High	6

76–100	Low	7
	High	8

**Table 2:** Radiographic union rates across the six treatment groups (10 weeks after surgery).

	Control	0.1 M	0.5 M	1.0 M	2.0 M	4.0 M
Full union	0/6 (0%)	0/6 (0%)	4/6 (66.7%)	5/6 (83.3%)	6/6 (100%)	6/6 (100%)
Partial union	1/6 (16.7%)	2/6 (33.3%)	1/6 (16.7%)	1/6 (16.7%)	0/6 (0%)	0/6 (0%)
Nonunion	5/6 (83.3%)	4/6 (67.7%)	1/6 (16.7%)	0/6 (0%)	0/6 (0%)	0/6 (0%)

Note. Control refers to the animals that received no endothelial progenitor cells (EPCs); 0.1 M, 0.5 M, 1.0 M, 2.0 M, and 4.0 M refer to the animals that received 1 × 10^5^, 5 × 10^5^, 1 × 10^6^, 2 × 10^6^, and 4 × 10^6^ EPCs, respectively.

## Data Availability

Data will be made available upon request to the authors.

## Supplementary Materials

Supplementary Materials Supplementary Table 1: Animal weights. Note: values are reported as mean (standard deviation). There are no statistically significant differences in animal weights between the groups (*p* > 0.05). Control refers to the animals that received no endothelial progenitor cells (EPCs); 0.1 M, 0.5 M, 1.0 M, 2.0 M, and 4.0 M refer to the animals that received 1 × 10^5^, 5 × 10^5^, 1 × 10^6^, 2 × 10^6^, and 4 × 10^6^ EPCs, respectively. Week 0 refers to the day of the surgery. Week 10 refers to the day animals were euthanized 10 weeks following the surgery. Supplementary Table 2: Radiographic scores. Note: values are reported as mean (standard deviation). ^*∗*^The 0.5 M, 1.0 M, 2.0 M, and 4.0 M groups are significantly different from the control group (*p* ≤ 0.05). ^#^The 0.5 M, 1.0 M, 2.0 M, and 4.0 M groups are significantly different from the 0.1 M group (*p* ≤ 0.05). Control refers to the animals that received no endothelial progenitor cells (EPCs); 0.1 M, 0.5 M, 1.0 M, 2.0 M, and 4.0 M refer to the animals that received 1 × 10^5^, 5 × 10^5^, 1 × 10^6^, 2 × 10^6^, and 4 × 10^6^ EPCs, respectively.
