# Supplementary material for: Endothelial Progenitor Cell Therapy for Fracture Healing: A Dose-Response Study in a Rat Femoral Defect Model
Source: J Tissue Eng Regen Med. 2023 Mar 9;2023:8105599. doi: 10.1155/2023/8105599 (PMC11918885; doi:10.1155/2023/8105599)
Supplement: Supplementary Materials — Supplementary Table 1: Animal weights. Note: values are reported as mean (standard deviation). There are no statistically significant differences in animal weights between the groups (p > 0.05). Control refers to the animals that received no endothelial progenitor cells (EPCs); 0.1 M, 0.5 M, 1.0 M, 2.0 M, and 4.0 M refer to the animals that received 1 × 10^5^, 5 × 10^5^, 1 × 10^6^, 2 × 10^6^, and 4 × 10^6^ EPCs, respectively. Week 0 refers to the day of the surgery. Week 10 refers to the day animals were euthanized 10 weeks following the surgery. Supplementary Table 2: Radiographic scores. Note: values are reported as mean (standard deviation). ∗The 0.5 M, 1.0 M, 2.0 M, and 4.0 M groups are significantly different from the control group (p ≤ 0.05). #The 0.5 M, 1.0 M, 2.0 M, and 4.0 M groups are significantly different from the 0.1 M group (p ≤ 0.05). Control refers to the animals that received no endothelial progenitor cells (EPCs); 0.1 M, 0.5 M, 1.0 M, 2.0 M, and 4.0 M refer to the animals that received 1 × 10^5^, 5 × 10^5^, 1 × 10^6^, 2 × 10^6^, and 4 × 10^6^ EPCs, respectively. [file 8105599.f1.docx]

**SUPPLEMENTARY TABLE 1** Animals weights

|  | **Control** | **0.1M** | **0.5M** | **1.0M** | **2.0M** | **4.0M** |
| --- | --- | --- | --- | --- | --- | --- |
| **Week 0 (g)** | 282 (9) | 281 (18) | 287 (18) | 273 (17) | 274 (8) | 276 (22) |
| **Week 10 (g)** | 350 (15) | 356 (19) | 355 (17) | 350 (18) | 356 (9) | 346 (36) |

*Note*: Values are reported as mean (standard deviation). There are no statistically significant differences in animal weights between the groups (*p* > 0.05). Control refers to the animals that received no endothelial progenitor cells (EPCs); 0.1M, 0.5M, 1.0M, 2.0M, and 4.0M refer to the animals that received 1x10^5^, 5x10^5^, 1x10^6^, 2x10^6^, and 4x10^6^ EPCs, respectively. Week 0 refers to the day of the surgery. Week 10 refers to the day animals were euthanized 10 weeks following the surgery.

**SUPPLEMENTARY TABLE 2** Radiographic scores

|  | **Control** | **0.1M** | **0.5M** | **1.0M** | **2.0M** | **4.0M** |
| --- | --- | --- | --- | --- | --- | --- |
| **2 weeks** | 1.75 (0.69) | 0.92 (0.20) | 2.17 (1.81) | 2.33 (2.36) | 3.25 (2.64) | 4.33 (2.94) |
| **4 weeks*^#^** | 1.83 (1.21) | 2.17 (1.21) | 4.83 (2.27) | 6.00 (1.58) | 6.83 (0.75) | 7.00 (0.63) |
| **6 weeks*^#^** | 2.50 (1.14) | 3.67 (1.81) | 6.25 (2.16) | 7.17 (0.82) | 7.08 (0.58) | 7.25 (0.27) |
| **8 weeks*^#^** | 2.50 (1.76) | 4.17 (2.09) | 6.75 (1.86) | 7.33 (0.93) | 7.58 (0.20) | 7.42 (0.38) |
| **10 weeks*^#^** | 2.50 (1.34) | 4.25 (2.12) | 6.92 (1.69) | 7.50 (1.00) | 7.67 (0.26) | 7.50 (0.00) |

*Note*: Values are reported as mean (standard deviation). * The 0.5M, 1.0M, 2.0M, and 4.0M groups are significantly different from the control group (*p* ≤ 0.05). ^#^ The 0.5M, 1.0M, 2.0M, and 4.0M groups are significantly different from the 0.1M group (*p* ≤ 0.05). Control refers to the animals that received no endothelial progenitor cells (EPCs); 0.1M, 0.5M, 1.0M, 2.0M, and 4.0M refer to the animals that received 1x10^5^, 5x10^5^, 1x10^6^, 2x10^6^, and 4x10^6^ EPCs, respectively.
